# Strahlenexposition bei häufigen interventionellen Eingriffen der Leber im Vergleich: Retrospektive Analyse von DeGIR-Registerdaten

**DOI:** 10.1007/s00117-020-00737-8

**Published:** 2020-08-20

**Authors:** Jonathan Nadjiri, Tobias Geith, Tobias Waggershauser, Lothar Heuser, Dominik Morhard, Arno Bücker, Philipp M. Paprottka

**Affiliations:** 1 Sektion für Interventionelle Radiologie, Klinikum rechts der Isar der Technischen Universität München, Ismaninger Straße 22, München, Deutschland; 2 Im Pastoratsbusch 49, Bochum, Deutschland; 3 Radiologie und Neuroradiologie, Leopoldina Krankenhaus Schweinfurt, Schweinfurt, Deutschland; 4 Klinik für Diagnostische und Interventionelle Radiologie, Universitätsklinikum der Universität des Saarlandes, Homburg /Saar, Deutschland

**Keywords:** Dosis, Leberinterventionen, Gallenweginterventionen, Transarterielle Chemoembolisation, DeGIR Registerdatenbank, Radiation monitoring, Liver interventions, Biliary interventions, Transcatheter arterial chemoembolization, DeGIR registry

## Abstract

**Hintergrund:**

Die transarterielle Chemoembolisation (TACE) oder auch Gallenganginterventionen stellen häufige Leberinterventionen dar.

**Ziel der Arbeit:**

In dieser retrospektiven Studie soll die Strahlenexposition der Patienten mit einem hepatischen Eingriff in Abhängigkeit von Art und Feinziel der Intervention analysiert und verglichen werden.

**Material und Methoden:**

Dies ist eine Analyse von 7003 DeGIR-Registerdatensätzen aus den Jahren 2016 bis 2018 für TACE und Gallenganginterventionen. Das Dosisflächenprodukt (DFP) und die Durchleuchtungszeit (DL) sowie die Interventionsart und das anatomisch definierte Feinziel der Interventionen wurden erfasst.

**Ergebnisse:**

Insgesamt lagen Dosiswerte für 4985 durchgeführte TACE und 2018 Gallenganginterventionen vor. Bei Gallenganginterventionen lag der Median des DFP bei 2594 (Interquartilbereich [IQR] = 1174–5858) cGy*cm^2^. Bei der TACE betrug der Median des DFP 11.632 [IQR = 5530–22.800] cGy*cm^2^ und lag damit signifikant höher als bei Gallenganginterventionen (*p* < 0,0001). Gallengangeingriffe mit dem höchsten DFP sind Interventionen am Ductus hepaticus, während Eingriffe mit der längsten DL an der Hepatikusgabel stattfinden.

**Diskussion:**

Die individuelle Strahlendosis für einen Patienten bei einer Leberintervention hängt weniger von der Komplexität des Eingriffs bzw. Durchleuchtungszeit ab, sondern von der Art des Eingriffs und vom Feinziel der Intervention. Die vorliegenden Dosisdaten können eine Hilfe sein, die Strahlenexposition bei einer Leberintervention bereits vor dem Eingriff grob abzuschätzen.

## Abstract (en)

**Background:**

Transcatheter arterial chemoembolization (TACE) and biliary interventions are common procedures.

**Objectives:**

In this retrospective study, the radiation exposure of patients undergoing hepatic intervention will be analyzed and compared depending on the type and objective of the intervention.

**Materials and methods:**

This is an analysis of 7003 data sets of performed TACEs and biliary interventions from the DeGIR registry for the years 2016, 2017, and 2018. The dose area product (DAP), fluoroscopy time (FT), type of intervention, and anatomically defined target were recorded.

**Results:**

Data with documented radiation doses were available for 4985 TACEs and for 2018 biliary interventions. For biliary interventions the median DAP was 2594 (interquartile range [IQR] = 1174–5858) cGycm^2^. For TACE, the median DAP was 11,632 [IQR = 5530–22,800] cGycm^2^ and significantly higher compared to biliary interventions (*p* < 0.0001). Biliary interventions with the highest DAP take place at the common hepatic duct; procedures with the longest FT were registered at the hepatic duct bifurcation.

**Conclusions:**

The individual radiation exposure during liver interventions is less dependent of the complexity of the procedure or the fluoroscopy time, but rather on the type of intervention and the anatomic target. The presented data can help to approximately estimate the radiation exposure in advance when planning an intervention.

Die Weiterentwicklung der interventionellen Radiologie mit Ausweitung der Indikationen, Verfügbarkeit und Patientenzahlen trägt zu einer zunehmenden Strahlenexposition von Patienten bei. Die transarterielle Chemoembolisation (TACE) und Gallenganginterventionen stellen häufige Eingriffe dar. Derzeit liegen keine genauen Daten darüber vor, welchen Einfluss technische Details wie z. B. die Art des Lebereingriffs, das genaue Ziel oder auch die Art der Interventionen auf die individuelle Strahlenexposition des Patienten haben. In dieser Studie soll die Strahlenexposition bei hepatischen Eingriffen in Abhängigkeit dieser technischen Details analysiert und verglichen werden.

## Hintergrund und Fragestellung

Weltweit ist die medizinisch bedingte durchschnittliche Strahlenexposition innerhalb der letzten Jahre trotz zahlreicher technischer Fortschritte zur Reduktion der Strahlenexposition stark angestiegen. Dies ist vor allem auf die zunehmende Anzahl an durchgeführten CT-Untersuchungen zurückzuführen [5]. Die Weiterentwicklung der interventionellen Radiologie hat zur Ausweitung der Indikationen, zu höherer Verfügbarkeit und einem damit verbundenen Anstieg der Patientenzahlen geführt. Somit hat die detaillierte Betrachtung der Strahlenexposition für dieses Anwendungsgebiet eine hohe Bedeutung. Sogar das zunehmende durchschnittliche Patientengewicht und das hierdurch bedingt größere zu durchstrahlende Volumen trägt zur allgemeinen Erhöhung der Strahlenexposition bei.

In der Regel bewegt sich die Strahlenexposition in der interventionellen Radiologie unterhalb der Schwelle für deterministische Schäden. Diese kann allenfalls in seltenen Ausnahmefällen überschritten werden.

Aufgrund der Strahlenexposition des Patienten und möglicher Folgen ist neben den differenzialtherapeutischen Überlegungen die Indikation der Untersuchung oder des Eingriffs genau abzuwägen und präzise zu stellen. Beim Eingriff selbst ist die Exposition für den Patienten nach dem ALARA-Prinzip so niedrig wie möglich zu halten („as low as reasonably achievable“; [2]). In Deutschland geben diagnostische Referenzwerte (DRW) dem Anwender eine Orientierung über die Effizienz des Strahlenschutzes im praktischen Alltag. Durch den Vergleich der für die einzelne Intervention festgestellten Strahlenexposition mit dem Referenzwert sollen Patienten vor zu hoher Exposition geschützt werden [7]. Die Erhebung von Dosisreferenzwerten ist in der Strahlenschutzverordnung (§ 125 Absatz 1 StrlSchV) und im Strahlenschutzgesetz (§ 185 Absatz 2 Nr. 2 StrlSchG) festgelegt.

Das Abdomen zählt aufgrund seiner großen epithelbesetzten Oberfläche zu einer strahlensensiblen Region des Körpers, welche jedoch häufig im Fokus von interventionell radiologischen Therapien steht [6, 8]. Dies ist z. B. bei einer transarteriellen Chemoembolisation (TACE) oder auch Gallenganginterventionen der Fall. Das Bundesamt für Strahlenschutz hat in seiner aktuellen Liste von 2018 für interventionell-radiologische Eingriffe am Erwachsenen lediglich für die TACE ein DFP von 23.000 cGy*cm^2^ als Dosisreferenzwert angegeben. Da die TACE in derselben anatomischen Region durchgeführt wird wie Gallenganginterventionen, könnte man in Ermangelung besserer Werte versucht sein, die DRW der TACE auf ebensolche Gallenganginterventionen anzuwenden. Derzeit liegen keine genauen Daten darüber vor, welchen Einfluss technische Details wie z. B. die Art des Eingriffs, also TACE im Vergleich zu Gallenganginterventionen, das genaue Ziel der Gallenganginterventionen oder auch die Art der Gallenganginterventionen, also Drainage, Bougierung etc., auf die individuelle Strahlenexposition des Patienten haben. Das Wissen über die mittlere Strahlenexposition bei Gallenganginterventionen und über den Einfluss von technischen Aspekten des Eingriffs auf die Strahlenexposition des Patienten könnte dem interventionellen Radiologen bei der Planung eines Eingriffs helfen.

Zielsetzung dieser Arbeit ist die Ermittlung von Strahlenexpositionswerten für verschiedene Gallenganginterventionen, deren Vergleich mit der TACE und die Ermittlung der Notwendigkeit für die Aufstellung von Referenzwerten.

## Studiendesign und Untersuchungsmethoden

### Einhaltung ethischer Standards

Diese retrospektive Analyse von Registerdaten steht im Einklang mit nationalem Recht sowie der Deklaration von Helsinki von 1975 (in der aktuellen, überarbeiteten Fassung). Die Daten wurden bis Ende 2018 gesammelt, liegen anonymisiert vor und wurden retrospektiv ausgewertet. Eine Patienteneinwilligung oder Zustimmung der lokalen Ethikkomission ist daher nicht erforderlich.

### Datenerfassung

Die Deutsche Gesellschaft für Interventionelle Radiologie und minimal-invasive Therapie (DeGIR) erfasst im Rahmen eines Qualitätssicherungsprogramms über ein Register vaskuläre und nichtvaskuläre Interventionen. Die Erfassung der Daten im Studienzeitraum erfolgte über Software von BQS (Institut für Qualität & Patientensicherheit GmbH) und Samedi über entsprechende Webportale und werden von der DeGIR für die vorliegende Studie freundlicherweise zur Verfügung gestellt. Die Dateneingabe zur Qualitätssicherung erfolgt auf freiwilliger Grundlage. Als Basis für die retrospektive Analyse wurden die anonymisierten DeGIR-Registerdaten aus den Jahren 2016, 2017 und 2018 für die TACE verwendet sowie die Daten aus dem Jahr 2017 und 2018 für Gallenganginterventionen. Die Erfassung der Dosis erfolgte über das registrierte Dosisflächenprodukt (DFP) in cGy*cm^2^. Zu dem erfolgte die Erfassung der Durchleuchtungszeit (DL) in Minuten. Folgende Interventionsarten wurden erfasst:

- selektive und superselektive TACE,
- Katheterbougierung, Ballondilatation und/oder Stentbehandlung von Gallengängen,
- interne und externe Katheterdrainagen von Gallengängen.

Die definierten Feinziele der Gallenganginterventionen waren folgende:

- rechter und linker Ductus hepaticus,
- Hepatikusgabel,
- Ductus hepaticus communis und Ductus hepatocholedochus,
- biliodigestive Anastomose und sonstige (z. B. Peripherie bei Leckage).

### Studienpopulation und Einschlusskriterien

Für die Jahre 2016, 2017 und 2018 standen insgesamt 14.080 Datensätze zur Verfügung. Zur Analyse wurden nur Datensätze aus Deutschland eingeschlossen. Datensätze ohne Dosisangaben wurden ausgeschlossen. Nach einer Plausibilitätsanalyse wurden nur Datensätze eingeschlossen, bei denen galt:

- Durchleuchtungszeit <360 min
- 250 cGy*cm^2^ < DFP < 100.000 cGy*cm^2^

**Figure 1:**
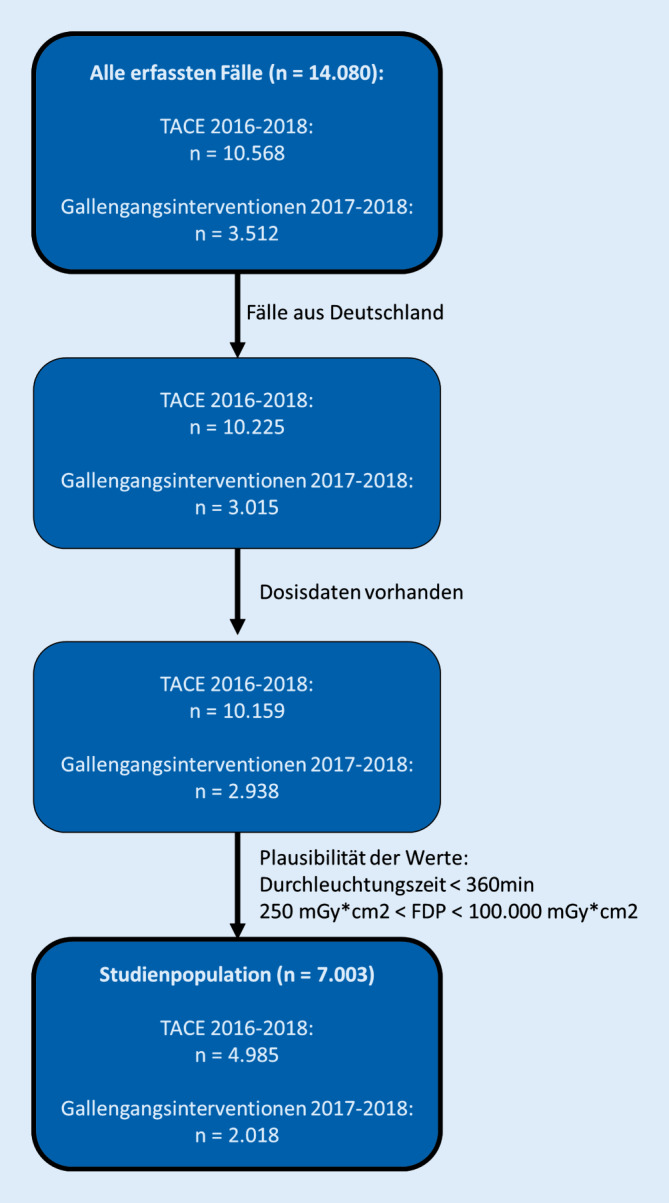

Eine Flowchart zur Darstellung der Einschlusskriterien und -zahlen ist in Abb. 1 illustriert.

Die gesamte Studienpopulation beträgt 7003 Patienten.

### Statistik

Zur deskriptiven Statistik wurde R Statistics (R version 3.5.3 [2019-03-11] – „Great Truth“) verwendet [9]. Die Normalverteilung wurde auf Basis der Histogramme angenommen. Zum Mittelwertvergleich der Gruppen wurde der Wilcoxon-Mann-Whitney-Test, zum Vergleich von mehr als zwei Gruppen eine Anova-Analyse durchgeführt. Zur Analyse von Korrelationen wurde eine lineare Regressionsanalyse mit Pearsons-Korrelationskoeffizienten durchgeführt. Als allgemeines Signifikanzniveau wurde *p* < 0,05 akzeptiert.

## Ergebnisse

### Galleninterventionen

**Figure 2:**
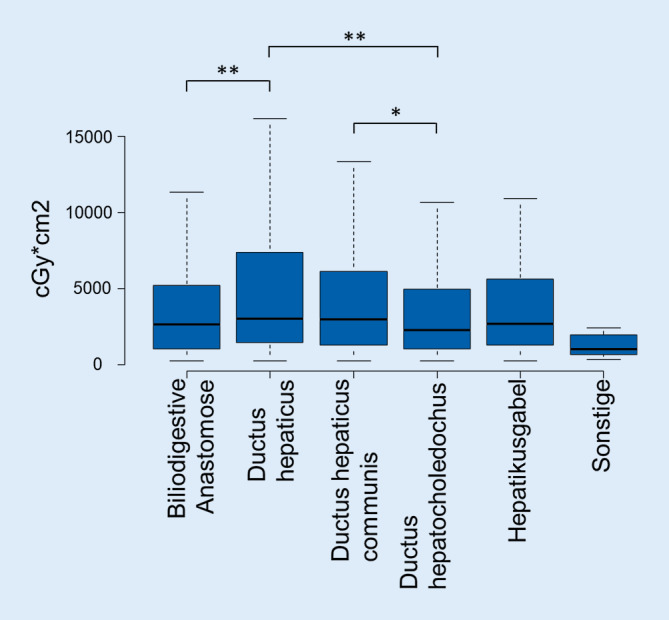

**Figure 3:**
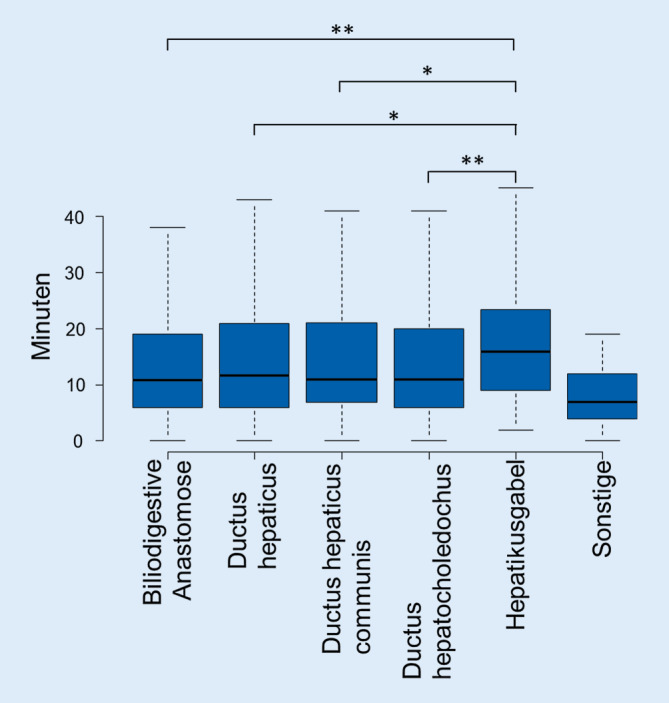

In den Jahren 2017 und 2018 wurden 2018 Gallenganginterventionen eingeschlossen. Nur für das Jahr 2017 liegt eine Kodierung der Interventionsart vor. 62 % der Patienten waren männlich. Das Durchschnittsalter lag bei 67 ± 13 Jahren. Eine Auflistung nach Feinzielen und Interventionsart ist in Tab. 1 angefügt. Bei Gallenganginterventionen lag der Median der DL bei 11 [IQR = 6–20] min; der Median des DFP lag bei 2594 [IQR = 1174–5858] cGy*cm^2^. Eine genaue Auflistung der DFP nach Feinziel ist in Abb. 2 dargestellt; die Abhängigkeit der DL vom Feinziel ist in Abb. 3 illustriert. Die längste DL haben Galleninterventionen an der Hepatikusgabel; diese ist signifikant höher als bei allen anderen Feinzielen. Das höchste DFP haben Interventionen am Ductus hepaticus; diese ist signifikant höher als Interventionen an einer biliodigestiven Anastomose und am Ductus hepatocholedochus. Die Korrelation zwischen DL und DFP ist gering, wenn auch hoch signifikant: R = 0,42; *p* < 0,0001.

**Table 1:** 

Eingriffe	2018
Durchschnittsalter in Jahren	67 ± 13
Geschlecht (männlich)	1264 (62 %)
*Interventionsarten (nur Jahr 2017)*	
Ballondilatation und/oder Stent	317 (24 %)
Katheterbougierung	31 (2 %)
Katheterdrainage	980 (74 %)
*Feinziel*	
Biliodigestive Anastomose	320 (16 %)
Ductus hepaticus	554 (27 %)
Ductus hepaticus communis	231 (11 %)
Ductus hepatocholedochus	788 (39 %)
Hepatikusgabel	95 (5 %)
Sonstige	30 (2 %)
Dosisflächenprodukt nach Interventionsart in cGy*cm^*2*^ *Median und Interquartil-Range in eckigen Klammern (nur 2017)*	
Ballondilatation und/oder Stent	3244 [IQR = 1363–6395]
Katheterbougierung	1816 [IQR = 1010–5367]
Katheterdrainage	2496 [IQR = 1138–5748]
Dosisflächenprodukt nach Feinziel in cGy*cm^*2*^ *Median und Interquartil-Range in eckigen Klammern*	
Biliodigestive Anastomose	2591 [1050–5207]
Ductus hepaticus	3041 [1448–7390]
Ductus hepaticus communis	2992 [1328–6140]
Ductus hepatocholedochus	2238 [1064–4957]
Hepatikusgabel	2687 [1300–5630]
Sonstige	983 [715–1962]
*Durchleuchtungszeit nach Interventionsart in Minuten* *Median und Interquartil-Range in eckigen Klammern (nur 2017)*	
Ballondilatation und/oder Stent	11 [6–21]
Katheterbougierung	7 [3–15]
Katheterdrainage	12 [6–20]
*Durchleuchtungszeit nach Feinziel in Minuten* *Median und Interquartil-Range in eckigen Klammern*	
Biliodigestive Anastomose	11 [6–19]
Ductus hepaticus	12 [6–21]
Ductus hepaticus communis	11 [7–21]
Ductus hepatocholedochus	11 [6–20]
Hepatikusgabel	16 [9–24]
Sonstige	7 [4–12]
*Median und IQR der Durchleuchtungszeit aller Gallengansinterventionen in Minuten*	11 [6–20]
Median und IQR des FDP aller Gallengansinterventionen in cGy*cm^*2*^	2594 [1174–5858]

### Transarterielle Chemoembolisation

**Figure 4:**
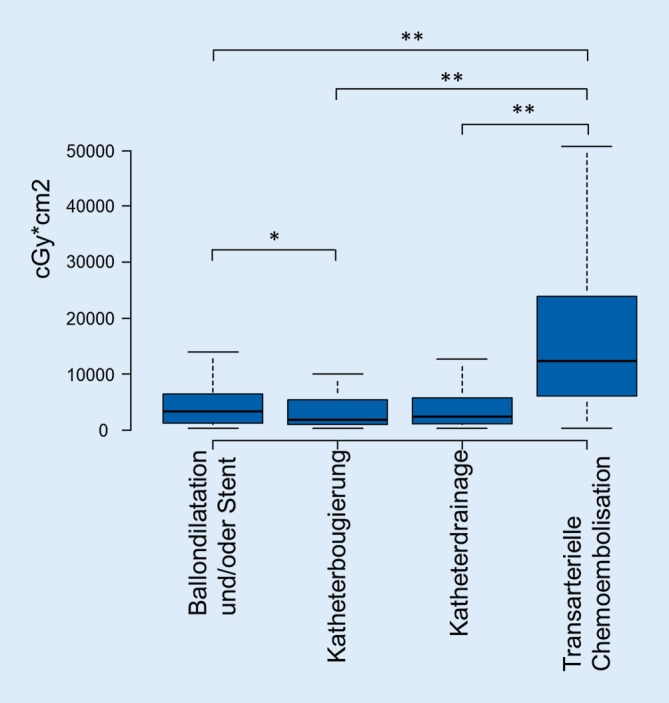

Aus den Jahren 2016, 2017 und 2018 wurden 4985 TACE-Eingriffe eingeschlossen. Das Durchschnittsalter lag bei 69 ± 10 Jahren; 3656 (73 %) der Patienten waren männlich. Eine Auflistung der DFP und DL ist in Tab. 2 aufgeführt. Bei der TACE betrug der Median der DL 16 [IQR = 10–25] min und lag damit signifikant über der DL der Gallenganginterventionen (*p* < 0,0001); der Median des DFP lag bei 11.632 [IQR = 5530–22.800] cGy*cm^2^ und damit ebenfalls signifikant höher als bei Gallenganginterventionen (*p* < 0,0001). Die Korrelation zwischen DL und DFP ist gering und hochsignifikant: R = 0,25; *p* < 0,0001. In Abb. 4 und 5 werden die Relationen des DFP und der DL in Abhängigkeit der Interventionsart illustriert.

**Table 2:** 

Eingriffe	4958
Durchschnittsalter in Jahren	69 ± 10
Geschlecht (männlich)	3656 (73 %)
Median und IQR des DFP aller TACE in cGy*cm^*2*^	11.632 [5530–22.800]
Median und IQR der Durchleuchtungszeit aller TACE in Minuten	16 [10–25]

**Figure 5:**
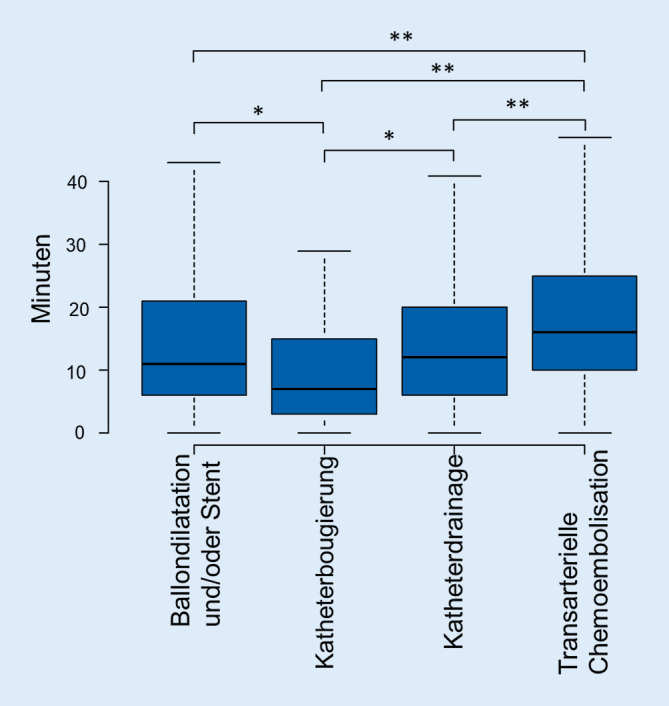

### Vergleich der Interventionsarten

Die TACE weist ein signifikant höheres DFP auf im Vergleich zur Ballondilatation und/oder Stentbehandlung, Katheterbougierung und Katheterdrainagen von Gallengängen, wobei die DL bei der TACE im Vergleich zur Ballondilatation und/oder Stentbehandlung von Gallengängen oder auch Katheterdrainagen von Gallengängen signifikant länger ist. Die Korrelation zwischen DL und DFP aller registrierter Leberinterventionen (TACE und Gallenganginterventionen) ist gering: R = 0,28; *p* < 0,0001.

## Diskussion

Neben möglichst hohen Erfolgsraten und niedrigen Komplikationsraten gehört auch eine möglichst geringe Strahlenexposition zu den wichtigen Qualitätszielen bei interventionell-radiologischen Eingriffen. Die Deutsche Gesellschaft für Interventionelle Radiologie (DeGIR) und die Deutsche Gesellschaft für Neuroradiologie (DGNR) betreiben erhebliche Anstrengungen zur Förderung der interventionellen Radiologie und bemühen sich in besonderem Maße darum, die Strahlenexposition von Patienten und Interventionalisten immer weiter zu reduzieren. Eine gut strukturierte theoretische und praktische Ausbildung ist hierzu eine Grundvoraussetzung. So haben die DeGIR und DGNR die Ausbildung auch jenseits der Facharztprüfung durch Zertifizierungsmaßnahmen für den einzelnen Radiologen, aber auch für institutionelle Ausbildungszentren eingeführt. Ein eigenes Curriculum wurde ebenso erstellt wie die Anforderungen an die praktische Weiterbildung für spezialisierte Radiologen über verschiedene Module hinweg klar definiert wurden [3]. Im Rahmen der Zertifizierung wird ein strukturiertes Ausbildungsprogramm mit speziellen Fortbildungskursen zur Verfügung gestellt, welches die Eingriffsqualität erhöhen kann. Ein flächendeckendes Angebot an minimal-invasiven Eingriffen kann somit zwar zu einer Zunahme der medizinischen Strahlenexposition insgesamt führen, gleichzeitig aber zu einer verbesserten individuellen Patientenversorgung beitragen. Als Werkzeug zur Verbesserung der Patientenversorgungsqualität dient u. a. das DeGIR-Register, welches zur klinikinternen Qualitätssicherung genutzt werden kann, aber zudem auch dem individuellen Radiologen zur Eingriffserfassung für die o. g. Zertifizierung dient.

Für Interventionen im Bereich der Leber liefert das Bundesamt für Strahlenschutz nur für die TACE ein DFP von 23.000 cGy*cm^2^ als Dosisreferenzwert. Weitere Interventionen wie die häufigen Gallengansinterventionen werden nicht eigens erwähnt, da dem BfS bislang hierfür keine Daten vorgelegen haben [7]. Im Median liegt das DFP für eine TACE in den Registerdaten unter dem vom BfS veröffentlichten DRW, allerdings wurde 2016, 2017 und 2018 in 1214 Fällen (17 %) eine Überschreitung der 2018 aktualisierten DRW basierend auf Daten aus 2016 und 2017 von 23.000 cGy*cm^2^ beobachtet. Da die Dosisreferenzwerte vom BfS auf der Basis der 75. Perzentilen berechnet werden, ist die Überschreitung in 17 % der Fälle eher positiv überraschend niedrig. Als wahrscheinlichster Grund dafür kommt die technische Weiterentwicklung der Eingriffsgeräte in Frage, welche zu einer geringeren Strahlenexposition führen und die Eingriffswerte dadurch deutlich unterhalb der Referenzwerte liegen können.

### Interventionsart

In Bezug auf die Gallenganginterventionen zeigt die DL einen positiven Zusammenhang mit der Komplexität des Eingriffs, soweit sich diese Komplexität aus dem anatomischen Ziel der Intervention ableiten lässt. Die Auswertung zeigt aber auch, dass bei einer TACE die höchste Strahlenexposition zu erwarten ist, etwa vierfach erhöht, obwohl die DL-Zeit nur etwa um ein Viertel höher ist als bei den Gallenganginterventionen. Ursächlich dafür ist die dosisintensivere digitale Subtraktionsangiographie (DSA) bei der TACE. Die Bildserien der DSA werden benötigt, um die Gefäßübersicht zu erstellen sowie die gefäßreichen Tumoren zu identifizieren und deren Tumorfeeder zu erkennen. Zur Kontrolle der Verteilung des Chemoembolisates werden dann in der Regel die Fluoroskopie und Einzelbilder genutzt. Insbesondere die Identifizierung von Feedergefäßen zum Tumor kann mehrmals erforderlich sein, wenn mehrere zu behandelnde Tumoren und/oder mehrere arterielle Tumorzuflüsse vorliegen. Zudem wird in einigen Zentren bei der TACE auch eine Rotationsangiographie (bzw. C‑Arm-CT) genutzt, aus deren Daten CT-ähnliche Bilder generiert werden. Diese Technik erhöht die Präzision und Sicherheit der superselektiven Tumortherapie, kann jedoch auch zur Erhöhung der Strahlenexposition führen [1]. Diese theoretische Erklärung der Strahlenexposition offenbart eine Schwäche der Qualitätssicherungssoftware, da in der vorliegenden Analyse der Jahre 2016 bis 2018 keine Informationen über die Verwendung einer C‑Arm-CT für die Interventionen vorliegen. In der aktuellen Version werden diese Informationen nun abgefragt, um in zukünftigen Analysen den angenommenen Zusammenhang zwischen der Verwendung der C‑Arm-CT und der Strahlenexposition untersuchen zu können.

Bei Gallenganginterventionen kommen hauptsächlich Fluoroskopie und Einzelbilder zum Einsatz, die gegenüber der DSA-Technik mit einer wesentlich geringeren Dosis auskommen.

### Feinziel der Intervention

Gallengangeingriffe mit dem höchsten DFP sind Interventionen am Ductus hepaticus, während die Eingriffe mit der längsten DL-Zeit an der Hepatikusgabel stattfinden. Dies unterstreicht noch einmal die Rolle einer empirischen Dosisauswertung in Abhängigkeit vom Feinziel der Intervention bzw. der Interventionsart, da die DL-Zeit allein nur ein schwacher Surrogatparameter für die Strahlenexposition des Patienten ist. Im Gegensatz dazu ist das Feinziel stärker mit der Strahlenexposition assoziiert und könnte dem interventionellen Radiologen zusammen mit den hier vorliegenden Daten vor dem Eingriff eine grobe Einschätzung der Strahlenexposition für den Patienten und mit größerer Einschränkung auch für den Interventionalisten geben.

Aufgrund theoretischer Überlegungen wäre zu erwarten, dass die Länge der DL-Zeit mit der Komplexität einer Gallengangintervention positiv korreliert. Somit wäre bei höherer Komplexität bzw. längerer DL-Zeit eine höhere Strahlenexposition zu erwarten; diese Theorie wird aber durch die vorliegenden Daten dieser Studie nicht bestätigt. Ein Grund könnte die Verwendung von Obliquen-Strahlengängen sein, welche für bestimmte Eingriffsarten bzw. Feinziele nötig sind. Das größere durchstrahlte Volumen führt bei obliquen Strahlengängen zu einer deutlichen Dosiserhöhung, obwohl die Komplexität bzw. die DL des Eingriffs durchschnittlich sind. Zusätzlich kann auch die Verwendung der DSA für bestimmte Eingriffe technisch bedingt unabhängig von der Komplexität des Eingriffs zu einer erhöhten Strahlenexposition führen; dies wurde in anderen Körperregionen bereits gezeigt [4, 10].

### Limitationen

Das Gewicht bzw. der Body-Mass-Index (BMI) der Patienten wurde bisher durch das DeGIR-Register nicht erfasst, sodass der Einfluss des durchstrahlten Weichgewebes und die konsekutive Justage der Belichtungsautomatik nicht explizit abschätzbar ist. Aufgrund der großen Anzahl der Patienten und der multizentrischen Daten kann der Effekt einer unterschiedlichen Beeinflussung durch den BMI auf einzelne Gruppen als gering erachtet werden. Registerdaten – insbesondere auf dem Boden freiwilliger Eingaben – unterliegen immer einem Selektionsbias; auch kann das Problem von fehlerhaften Eingaben nur begrenzt beeinflusst werden. Um die Auswirkungen dieser Nachteile zu verringern, wurden Extreme aus der Datenanalyse ausgeschlossen, was dann aber wiederum jenseits der Verringerung der Anzahl der Datensätze den Effekt eines Bias erhöht.

## Fazit für die Praxis

- Die individuelle Strahlenexposition für einen Patienten bei einer Leberintervention hängt weniger von der Komplexität des Eingriffs oder der Durchleuchtungszeit, sondern vielmehr von der Art des Eingriffs und vom Feinziel der Intervention ab.
- Eine TACE ist mit einer signifikant höheren Strahlenexposition für den Patienten assoziiert als Gallenganginterventionen.
- Der Grund für die unterschiedlichen Dosiswerte ist wahrscheinlich die Verwendung bestimmter Techniken, wie die digitalen Subtraktionsangiographie oder Oblique-Strahlengänge, die vermehrt bei bestimmten Interventionsarten oder anatomischen Feinzielen zum Einsatz kommen.
- Grenzwerte für die TACE eignen sich nicht als Referenz für Gallenganginterventionen.
